# Persistent inpatient delirium associated with increased length of stay and mortality

**DOI:** 10.1371/journal.pone.0331245

**Published:** 2025-09-02

**Authors:** Jerry Bradley, Fei Tang, Zachary Panzarella, Jacob Nanney, Iriana Hammel, Bill Bryant, Darby Cole

**Affiliations:** 1 Department of Medicine, University of Miami Miller School of Medicine, Miami, Florida, United States of America; 2 Miami Veterans Administration (VA) Healthcare System Geriatric Research, Education, and Clinical Center (GRECC), Miami, Florida, United States of America; 3 Department of Family and Community Medicine, University of Louisville, Owensboro, Kentucky, United States of America; Hospital Sirio-Libanes, BRAZIL

## Abstract

**Introduction:**

Delirium is associated with an increased risk of post-hospitalization mortality. However, the impact of persistent delirium on mortality is not well defined.

**Methods:**

We conducted a retrospective cohort study on the association of non-persistent or persistent delirium and mortality. We included patients aged > 65 years admitted to the Owensboro Health Regional Hospital between August 2021 and August 2022. Delirium was determined based on a score of ≥ 2 on the Nurse Delirium Screening Scale (NuDESC) recorded on all admitted patients three times a day during nursing shift change. Multivariate logistic regression models were used to evaluate the association between non-persistent or persistent delirium and 30-day, 60-day, 90-day, 180-day, and 360-day mortality after adjusting for covariates. Sensitivity analysis was performed to compare different definitions of delirium as ≥ 2, ≥ 3, or ≥ 4 days of delirium occurring consecutively or non-consecutively.

**Results:**

4560 hospitalized patients were included in this study. Of these, 634 (13.9%) were identified as having delirium (persistent or non-persistent) and 3926 (86.1%) as without delirium. Patients with delirium were slightly older than those without (77 ± 7.9 vs. 79.5 ± 8.5 years old). The patients in each group were relatively comparable in terms of sex, race, smoking status, Medicare user status, and comorbidities. After adjusting for comorbidities, delirium was associated with an increase of mortality at 30 days for patients with persistent delirium of 2 days (aOR 4.88, 95% CI 3.63–6.54), 3 days (aOR 5.64, 95% CI 3.95–7.98), 4 days (aOR 7.21, 95% CI 4.74–10.8). Persistent delirium was associated with higher 60-day, 90-day, 180-day, and 360-day mortality rates, with an incremental increase in the risk of mortality for each additional day of delirium.

**Conclusion:**

Persistent delirium is consistently associated with increased mortality, with an increased risk of mortality for each additional day of delirium, underscoring the need for early identification and treatment.

## Introduction

Delirium is a complex medical condition driven by multiple risk factors [1,2]. Several tools have been developed to help clinicians diagnose and recognize this condition in inpatient settings [3]. Delirium is associated with increased mortality in the emergency department (ED) [4], intensive care unit (ICU) [5], and inpatient care settings [6]. Significant issues related to screening remain, particularly pertinent to staff workload and cultural barriers [7,8]. The impact of delirium on mortality after discharge can extend up to one year [9,10]. A growing body of research has attempted to evaluate the effects of delirium duration or persistent delirium on mortality.

Persistent delirium is not clearly defined and depends on the clinical setting. One definition of persistent delirium refers to patients who develop delirium during hospitalization and do not fully return to their baseline until the time of discharge or for up to several months after discharge [11]. This can be observed in the post-acute care environment, often driven by the presence of other geriatric syndromes [12]. A recent systematic review and meta-analysis attempted to understand the impact of this definition of persistent delirium on clinical outcomes. The authors noted several limitations with the current evidence reporting differences in the way delirium was determined, the number of time points in which delirium was assessed, and the extent to which delirium was followed up post-discharge [13]. These differences in reporting standards have made it difficult to compare the outcomes between studies during the post-discharge period.

Another definition of persistent delirium is based on the number of days the patient was documented to be delirious while hospitalized. Much of the literature is found in the ICU setting, where delirium screening is more common. One study reported that persistent delirium may be defined as delirium that begins in the ICU and continues after a patient is transferred to the general floor [14]. Another study defined it as having a period of 4 days of delirium by screening, either consecutively or non-consecutively [15]. These studies further underline the lack of consensus regarding the definition of persistent delirium in acute-care settings.

In order to simplify this approach, our study was designed to understand the impact of persistent delirium on mortality and length of stay by analyzing the number of days a patient spent in delirium while hospitalized. Keeping methodologically similar to prior studies, persistent delirium was counted as the number of days in delirium occurring consecutively or non-consecutively [15]. Due to the retrospective design, our study was not able to address issues with having delirium on discharge or post-discharge from the hospital in regard to these clinical outcomes. The objective of this study is to determine if there is a clinically and statistically significant cut off point for the number of days in persistent delirium for mortality and length of stay. We hypothesize that patients with persistent delirium will have a longer length of stay and mortality than patients with non-persistent delirium and those without delirium.

## Methods

### Delirium screening and determination

The Owensboro Health Regional Hospital and the University of Louisville implemented the Inpatient Delirium Reduction and Early Acute Management (iDREAM) Process Improvement Initiative in 2021. The Nursing Delirium Screening Scale (NuDESC) was used to screen the patients for delirium. Screening was performed at each nursing shift change, three times per day, for the duration of the inpatient admission. Scores were recorded into the electronic medical record system. A score ≥ 2 was considered positive for delirium. A single positive value for the day was considered as delirium-positive, even if the other two scores were negative. Given the waxing and waning nature of delirium, we could not retrospectively determine which patients may have remained delirious, even though their NuDESC scores may have returned to baseline for a day before another documented positive delirium screening. Patients were considered to be persistently delirious if they had multiple positive NuDESC scores on consecutive or non-consecutive days, at any time during hospitalization.

### Statistical analysis

We conducted a retrospective cohort study of patients aged > 65 years admitted to the Owensboro Health Regional Hospital between August 2021 and December 2023. Delirium was determined based on a score of ≥ 2 on the Nurse Delirium Screening Scale (NuDESC) recorded on all admitted patients three times a day during nursing shift change. Continuous variables (age, length of stay) are presented as mean±standard deviation and categorical variables (sex, race, smoking status, diabetes, hypertension, dementia, COPD, obesity, heart disease, and heart failure) as frequencies and percentages. Multivariate logistic regression models were used to compare the odds of mortality for non-persistent delirium and persistent delirium with the odds of mortality for patients without delirium. Variable multicollinearity was examined by VIF. Model fitting for the logistic regression were checked by Hosmer-Lemeshow tests. For sensitivity analysis, we defined persistent delirium as at least two days of delirium, at least three days of delirium, and at least four days of delirium. We used 30-day, 60-day, 90-day, 180-day, 360-day mortality rates for the model, adjusting for age, sex, race, smoking status, diabetes, hypertension, dementia, COPD, obesity, heart failure, and heart disease. Multivariate linear regression was used to evaluate the association between delirium and the length of hospital stay. Statistical analysis was performed using R (R project for statistical computing, version 4·0·5).

### Primary outcomes

The primary outcomes of the study were mortality at 30, 60, 90, 180, and 360 days for each group of patients with and without COVID-19 or Delirium. The length of stay was an additional outcome.

### IRB approval

This retrospective study was approved by the University of Louisville Institutional Review Board (IRB). The study was additionally approved by the Owensboro Health Regional Hospital Research Review Committee, with oversight and coordination provided by the Owensboro Health Department of Quality Management. The IRB granted a waiver for informed consent. The data was fully anonymized according to the University of Louisville and Owensboro Health research standards before analysis. The data was accessed from 1/12/2025 until 5/7/2025.

## Results

### Baseline characteristics

A total of 4560 hospitalized patients were included in the study, 261 (5.7%) of whom had one day of delirium, 373 (8.2%) had at least two days of delirium, and 3926 (86.1%) did not. Patients without delirium were slightly younger than those with only one day or at least two days of delirium (77.0 ± 7.6 years old v.s. 79.5 ± 8.5, 79.5 ± 8.6). Among the delirium-negative patients, 1871 (47.7%) were male, and 3782(96.3%) were white or Caucasian. Among patients with only one day of delirium, 135 (51.7%) were male and 253 (96.9%) were white or Caucasian. Lastly, in patients with at least two days of delirium, 171 (45.8%) were male and 362 (97.1%) were white or Caucasian. More than half of the patients were Medicare users, 2254 (57.4%) for delirium-negative patients, 168 (64.4%) for patients with one day of delirium, and 221 (59.2%) for patients with two or more days of delirium.

### Association of delirium and dementia

Patients with at least two days of delirium had a higher dementia rate (32 (8.6%)) compared to non-persistent delirium patients (12 (4.6%)) or patients without delirium (70 (1.8%)) (p < 0.001). The three groups had similar rates of obesity, diabetes, hypertension, COPD, heart failure, and heart disease.

### Persistent delirium and length of stay

Persistent delirium patients had a longer length of stay than non-persistent delirium patients or non-delirium patients (9.5 ± 10.9 days v.s. 5.3 ± 5.2 days and 7.2 ± 7.3 days) (p < 0.001).

### Association of persistent delirium with mortality

**Fig 1:**
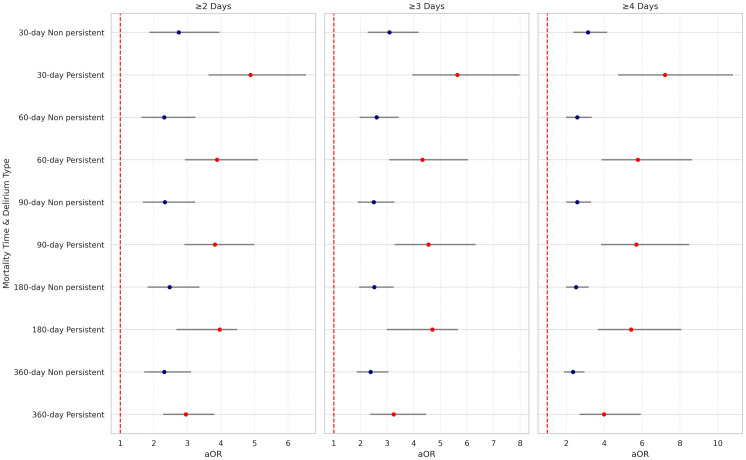
Forest plot of adjusted odds ratios for mortality by time Interval and number of days in persistent delirium.

**Fig 2:**
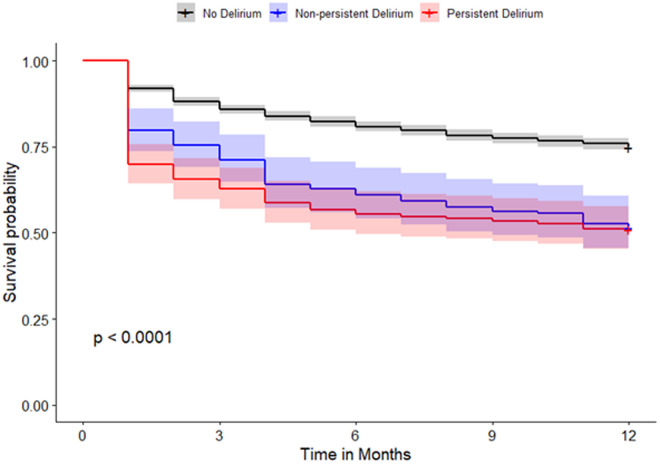
Survival plot comparing non-persistent and persistent delirium in patients without delirium for patients with persistent delirium defined as ≥ 2 days of delirium.

**Fig 3:**
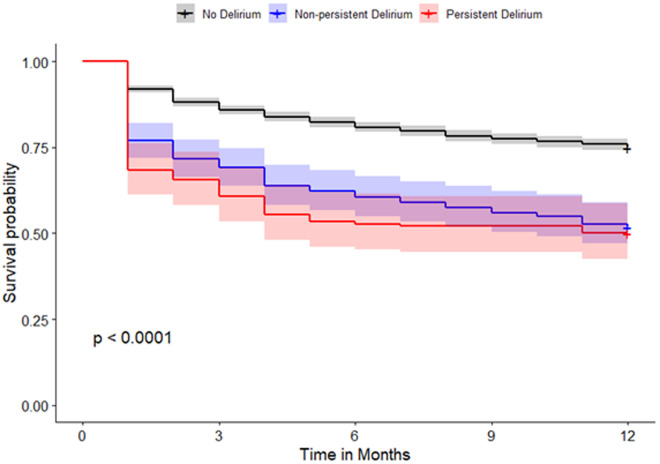
Survival plot comparing non-persistent and persistent delirium in patients without delirium for patients with persistent delirium defined as ≥ 3 days of delirium.

**Fig 4:**
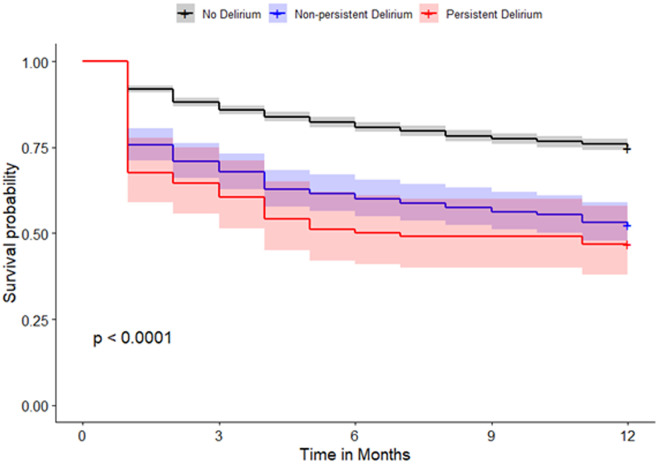
Survival plot comparing non-persistent and persistent delirium in patients without delirium for patients with persistent delirium defined as ≥ 4 days of delirium.

The 30-day, 60-day, 90-day, 6-month, and one-year mortality rates were highest in patients with persistent delirium, followed by patients with non-persistent delirium and those without delirium (Table 1). When persistent delirium is defined as at least three days of delirium, it was associated with an increase in the odds of death at 30 days post-hospitalization of 464% (aOR=5.64, 95% CI: 3.95–7.98), 233% at 60 days (aOR=4.33, 95% CI: 3.08–6.04) 356% at 90 days (aOR=4.56, 95% CI: 3.28–6.32) 370% at 180 days (aOR=4.70, 95% CI: 2.99–5.67) and lastly 225% increase in the odds of death within 360 days of hospitalization (aOR=3.25, 95% CI: 2.37–4.47). These odds ratios were compared to those of patients without delirium and adjusted for age, sex, race, smoking status, diabetes, hypertension, dementia, COPD, obesity, heart disease, and heart failure. Although the estimated 95% CIs for persistent delirium overlapped with those for non-persistent delirium, persistent delirium presented higher odds of mortality than non-persistent delirium (Table 2, Figs 1–4,). Similar results were observed when persistent delirium was defined as at least two days of delirium or at least four days of delirium ( Table 2 , Figs 1–4,).

**Table 1:** Characteristics of patients without delirium, one day of delirium, or at least two days of delirium.

	Delirium Negative N = 3926 (86.1%)	1 day of delirium N = 261 (5.7%)	≥ 2 days of delirium N = 373 (8.2%)	P-values
**Age**	77.0 ± 7.9 (76.0)	79.5 ± 8.5 (80.0)	79.5 ± 8.6 (79.0)	<0.001
**Male Gender**	1871 (47.7%)	135 (51.7%)	171 (45.8%)	0.331
**Race**				
** White or Caucasian**	3782 (96.3%)	253 (96.9%)	362 (97.1%)	0.998
** Black or African American**	120 (3.1%)	8 (3.1%)	9 (2.4%)	
** Hispanic/Latin American**	6 (0.2%)	0 (0.0%)	1 (0.3%)	
**Smoking Status**				
** Everyday**	378 (12.8%)	23 (11.3%)	39 (13.7%)	0.002
** Former**	1455 (47.9%)	81 (39.7%)	126 (44.2%)	
** Never**	1099 (36.2%)	91 (44.6%)	106 (37.2%)	
** Medicare User**	2254 (57.4%)	168 (64.4%)	221 (59.2%)	0.999
**Comorbidity**				
** Dementia**	70 (1.8%)	12 (4.6%)	32 (8.6%)	<0.001
** Diabetes**	310 (7.9%)	20 (7.7%)	30 (8.0%)	0.985
** Hypertension**	791 (20.1%)	53 (20.3%)	71 (19.0%)	0.872
** COPD**	453 (11.5%)	32 (12.3%)	42 (11.3%)	0.923
** Obesity**	375 (9.6%)	22 (8.4%)	28 (7.5%)	0.378
** Heart Disease**	410 (10.4%)	28 (10.7%)	41 (11.0%)	0.940
** Heart Failure**	611 (15.6%)	34 (13.0%)	44 (11.8%)	0.095
** Length of Stay (Days)**	5.3 ± 5.2 (4.0)	7.2 ± 7.3 (6.0)	9.5 ± 10.9 (7.0)	<0.001
**Mortality**				
** 30 days**	338 (8.6%)	55 (21.1%)	111 (29.8%)	<0.001
** 60 days**	480 (12.2%)	65 (24.9%)	126 (33.8%)	<0.001
** 90 days**	565 (14.4%)	76 (29.1%)	140 (37.5%)	<0.001
** 180 days**	767 (19.5%)	99 (37.9%)	164 (44.0%)	<0.001
** 360 days**	996 (25.4%)	119 (45.6%)	185 (29.6%)	<0.001

**Table 2:** Mortality by time interval and number of days in persistent delirium.

	Death #	aOR	Death #	aOR	Death #	aOR
	≥ 2 days of delirium		≥ 3 days of delirium		≥ 4 days of delirium	
30-day Mortality						
No Delirium	384 (9.2%)	Reference	384 (9.2%)	Reference	384 (9.2%)	Reference
Non persistent Delirium,	68 (23.5%)	2.74 (1.87–3.95)	108 (24.5%)	3.09 (2.27–4.18)	134 (25.4%)	3.15 (2.37–4.16)
Persistent Delirium	126 (30.8%)	4.88 (3.63–6.54)	86 (33.3%)	5.64 (3.95–7.98)	60 (35.3%)	7.21 (4.74–10.8)^***^
60-day Mortality						
No Delirium	535 (12.8%)	Reference	535 (12.8%)	Reference	535 (12.8%)	Reference
Non persistent Delirium,	78 (27.0%)	2.31 (1.63–3.24)	125 (28.4%)	2.61 (1.97–3.44)	152 (28.8%)	2.58 (1.98–3.34)
Persistent Delirium	141 (34.5%)	3.88 (2.93–5.10)	94 (36.4%)	4.33 (3.08–6.04)	67 (39.4%)	5.78 (3.86–8.62)^***^
90-day Mortality						
No Delirium	629 (15.1%)	Reference	629 (15.1%)	Reference	629 (15.1%)	Reference
Non persistent Delirium,	89 (30.8%)	2.33 (1.67–3.23)	138 (31.4%)	2.50 (1.90–3.27)	170 (32.2%)	2.58 (2.00–3.31)
Persistent Delirium	156 (38.1%)	3.82 (2.91–4.99)	107 (41.5%)	4.56 (3.28–6.32)^***^	75 (44.1%)	5.70 (3.83–8.49)^***^
180-day Mortality						
No Delirium	839 (20.1%)	Reference	839 (20.1%)	Reference	839 (20.1%)	Reference
Non persistent Delirium,	114 (39.4%)	2.47 (1.81–3.36)	171 (38.9%)	2.52 (1.95–3.25)	207 (39.2%)	2.52 (1.99–3.18)
Persistent Delirium	181 (44.3%)	3.96 (2.67–4.49)	124 (48.1%)	4.70 (2.99–5.67)	88 (51.8%)	5.42 (3.66–8.07)^***^
360-day Mortality						
No Delirium	1076 (25.8%)	Reference	1076 (25.8%)	Reference	1076 (25.8%)	Reference
Non persistent Delirium,	135 (46.7%)	2.31 (1.71–3.11)	204 (46.4%)	2.38 (1.86–3.05)	245 (46.4%)	2.36 (1.88–2.96)
Persistent Delirium	203 (49.6%)	2.95 (2.28–3.80)	134 (51.9%)	3.25 (2.37–4.47)	93 (54.7%)	3.99 (2.70–5.94)

*** Indicates where the confidence intervals of persistent delirium do not overlap with those of persistent delirium.

When persistent delirium is defined as at least four days of delirium, it showed a 621% increase in the odds of death within 30 days post-hospitalization (aOR=7.21, 95% CI: 4.74–10.8) 478% at 60 days (aOR=5.78, 95% CI: 3.86–8.62); 470% at 90 days (aOR=5.70, 95% CI: 3.83–8.49); 442% at 180 days (aOR=5.42, 95% CI: 3.66–8.07); and lastly 299% increase in the odds of death within 360 days of hospitalization (aOR=3.99, 95% CI: 2.70–5.94). These odds ratios were compared to those of patients without delirium and adjusted for age, sex, race, smoking status, diabetes, hypertension, dementia, COPD, obesity, heart disease, and heart failure. Persistent delirium at 4 days showed increased odds of mortality compared to non-persistent delirium with non-overlapping 95% CIs (Table 2, Figs 1–4).

## Discussion

To our knowledge, this is one of the first studies to examine the effects of persistent delirium on mortality outcomes in hospitalized patients. Our findings demonstrate that patients who develop persistent delirium have an increased length of stay and mortality compared to those who do not develop delirium. These findings are consistent with other studies showing that delirium increases the risk of mortality [9]. However, our study demonstrates that it is not only the presence of delirium but also the total number of days spent in delirium that affects post-hospitalization mortality up to one year.

### Defining persistent delirium

Our findings show that the greater the total number of days a patient is delirious, the greater the risk of mortality post-discharge. In this study, we defined persistent delirium based on the total number of occurrences, either consecutively or non-consecutively, during hospitalization. At our hospital, delirium was assessed using the Nursing Delirium Screening Scale (NuDESC) at each nursing shift change that occurred three times a day. The NuDESC is a well-validated tool for detecting delirium, with a score of 2 or more as positive [16].

Given the uncertainty surrounding the definition of persistent delirium in terms of total days delirious, our study analyzed three common time points: two, three, and four days of delirium. Based on the adjusted odds ratio, having delirium for at least 2 and 3 days is associated with an increased risk of mortality. Though not all the differences were statistically significant, a clear trend was observed, particularly concerning longer delirium duration. Even though four-or-more-day delirium was most clearly linked with death, we did find rising risk from as early as two days, and thus persistent delirium cannot be defined by any single threshold. In all cases, the mortality risk after discharge remained elevated for up to one year for each persistent delirium category (Fig 1).

From a purely statistical perspective, having delirium for four or more days would be a distinguishing cutoff in terms of clinical outcomes. However, from a clinical perspective, there is a trend for increased mortality rates beginning at two days with each additional day of delirium increasing that risk. Therefore, it is challenging to draw a line between what is and is not persistent delirium based on the total number of days by mortality alone. Given these trends in the data, the findings support that persistent delirium in this context should be defined as having delirium either consecutively or non-consecutively for two or more days. Further research is required to determine the degree to which clinical outcomes are affected by persistent delirium, and the total number of days in that state. Persistent delirium, as defined in this study, may have important implications for future quality benchmarks. These results support the need for urgent and proactive interventions to reduce the incidence of delirium as well as the total number of days spent in delirium.

### Limitations

One of the strengths of our study was our ability to identify delirium on a daily basis. As delirium scores were determined for each shift, we were able to have several screening points to ensure that delirium was appropriately captured and trended during hospitalization. This allowed us to accurately determine the total number of days a patient was delirious without relying on a review of clinical documentation and ICD-10 codes. One of the limitations of our study is that we could not determine which patients maintained persistent delirium once they were discharged. In our study, we defined delirium based on the total number of days the patient was delirious while in the hospital. Given the retrospective nature of our study, we could not determine whether patients remained delirious when they were discharged and how long they were delirious post-discharge. Because of this, our mortality data may be skewed, as patients may have been persistently delirious post-discharge, which may have potentially increased their risk of mortality. Therefore, it was difficult to draw conclusions regarding the association between delirium and mortality in this cohort. Another limitation of our study was related to the screening method used. Although the NuDESC tool is well validated, several studies have highlighted the need for additional changes in the definition of a positive result to improve the sensitivity of patients who may be delirious [17,18]. In our study, we chose to represent delirium-positive patients, consistent with the original scale. Therefore, it is possible that the prevalence and duration of delirium may be underreported. Misclassification bias may have occurred due to variability in nursing staff application of the NuDESC tool. All nurses were trained to use this tool. Because data was collected three times a day by three separate nurses the probability of misclassification was likely diminished as delirium was considered positive if only one screen was positive for the entire day. Lastly, our study attempted to control for common risk factors for delirium and mortality. However, we could not adjust the model for all potential cofounding factors.

## Conclusion

Persistent delirium is associated with increased mortality and increased length of hospital stay. Further research is required to determine how the total number of days of persistent delirium affects clinical outcomes.
